# The Development of a Canine Anorectal Autotransplantation Model Based on Blood Supply: A Preliminary Case Report

**DOI:** 10.1371/journal.pone.0044310

**Published:** 2012-09-06

**Authors:** Jun Araki, Yuji Nishizawa, Tatsuo Nakamura, Tomoyuki Sato, Munekazu Naito, Satoshi Fujii, Makoto Mihara, Isao Koshima

**Affiliations:** 1 Department of Plastic Surgery, University of Tokyo, Tokyo, Japan; 2 Department of Bioartificial Organs, Institute for Frontier Medical Science, Kyoto University, Kyoto, Japan; 3 Saitama Shinkaibashi Clinic, Saitama, Japan; 4 Department of Anatomy, Tokyo Medical University, Tokyo, Japan; 5 Pathology Division, Research Center for Innovative Oncology, National Cancer Center Hospital East, Chiba, Japan; University of Colorado, United States of America

## Abstract

Colostomy is conventionally the only treatment for anal dysfunction. Recently, a few trials of anorectal transplantation in animals have been published; however, further development of this technique is required. Moreover, it is crucial to perform this research in dogs, which resemble humans in anorectal anatomy and biology. We designed a canine anorectal transplantation model, wherein anorectal autotransplantation was performed by anastomoses of the rectum, inferior mesenteric artery (IMA) and vein, and pudendal nerves. Resting pressure in the anal canal and anal canal pressure fluctuation were measured before and after surgery. Graft pathology was examined three days after surgery. The anal blood supply was compared with that in three beagles using indocyanine green (ICG) fluorescence angiography. The anorectal graft had sufficient arterial blood supply from the IMA; however, the graft’s distal end was congested and necrotized. Functional examination demonstrated reduced resting pressure and the appearance of an irregular anal canal pressure wave after surgery. ICG angiography showed that the pudendal arteries provided more blood flow than the IMA to the anal segment. This is the first canine model of preliminary anorectal autotransplantation, and it demonstrates the possibility of establishing a transplantation model in dogs using appropriate vascular anastomoses, thus contributing to the progress of anorectal transplantation.

## Introduction

Defecation is a major activity of daily living. It may be impaired because of congenital anal dysfunction caused by anal atresia or Hirschprung’s disease, intractable anal fistula, and rectal or perianal cancer resections. In most of these cases, colostomy is the only treatment.

Following publication of the pioneering report on abdominoperineal resection of the rectum by the British surgeon Miles in 1908, colostomy gained international acclaim throughout the 20th century [1], [2]. The number of patients who currently undergo colostomy amounts to approximately 200,000 in Japan and 700,000 in the U.S.A. Although this procedure is essential to preserve the patients’ life, it causes a lot of problems such as troublesome postoperative management, cosmetic concerns, and psychological distress. In fact, some patients prefer death to living with a stoma [3]. In addition, psychosocial sequelae are inevitable in patients who undergo ostomies [2], [4]. To address these issues, gracilis [5] and gluteus maximus muscle transfer [6] and artificial sphincter implantation [7] have been reported for restoring anal function. However, none of these is a gold standard technique because anal functions are very complicated [8]. Meanwhile, owing to improvements in surgical instruments and immunosuppressive therapy, organ transplantation has gained great popularity worldwide. Allotransplantation of not only vital organs but also the face [9], larynx [10], trachea [11], and the upper [12] and lower extremities [13] is being performed to improve patients’ quality of life. Therefore, reconstructing tissues with complicated functions, such as the face and hands, has become possible [14], [15].

Allotransplantation of the anorectal segment (all organs involved in defecation, including the perineal skin, anus, rectum, and the sphincter muscle) has strong potential as an innovative therapy for anal dysfunction. Anal transplantation models using rats [16]–[18] and swine [19] have been reported in experimental trials; however, these experimental systems have considerable drawbacks. Because rats and pigs do not have the capacity to inhibit defecation and have habits similar to those of humans, they are not particularly useful as model subjects for human application. Dogs living with humans and having defecation control are the most suitable of all laboratory animals for anal function evaluation. Therefore, we performed anorectal autotransplantation in beagle dogs in this study.

**Figure 1:**
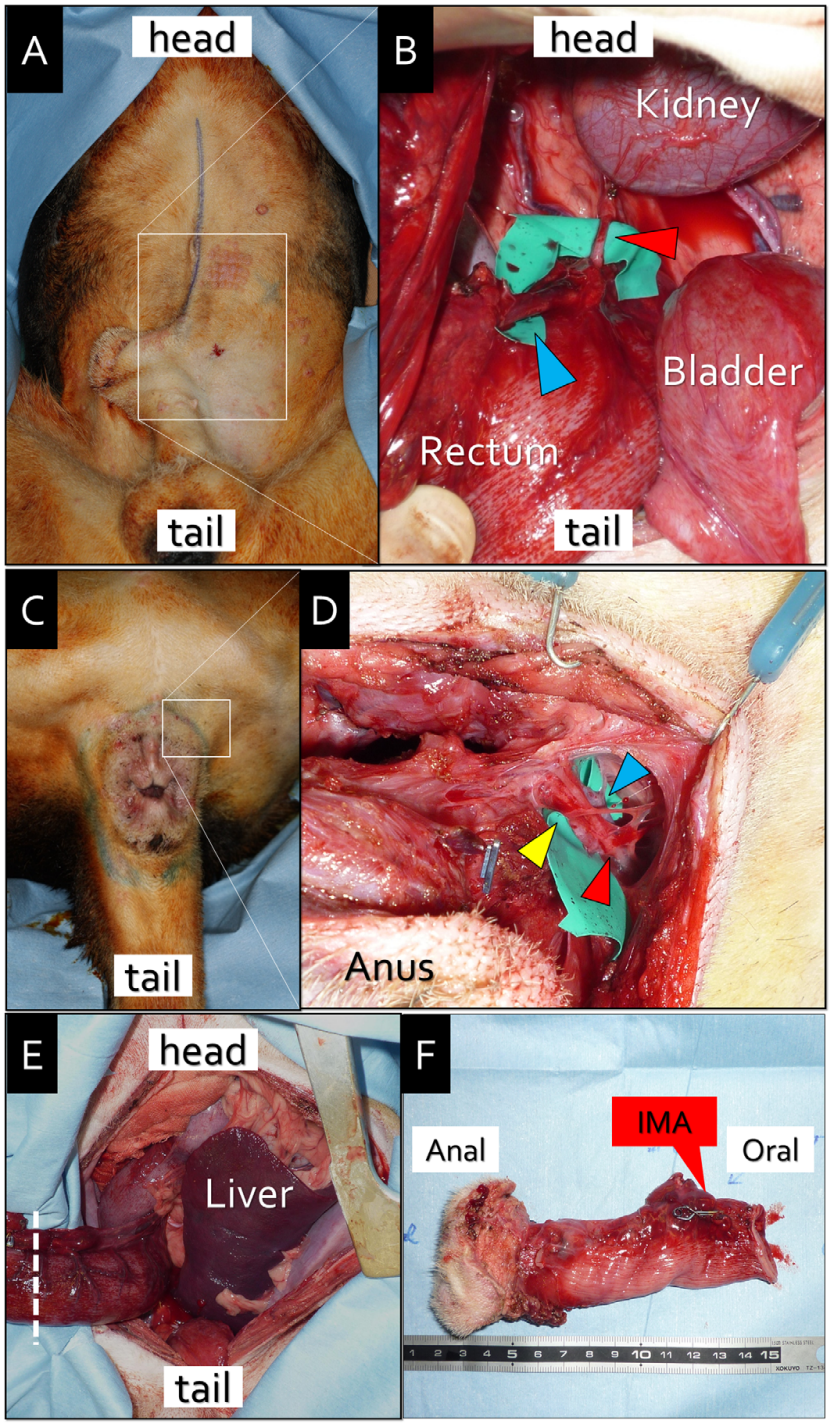
Anorectal graft harvesting. A. Median laparotomy. B. Intraperitoneal view of the inferior mesenteric artery (red arrowhead) and inferior mesenteric vein (blue arrowhead). C. Circumanal incision. D. Perineal view of the left pudendal artery (red arrowhead), pudendal vein (blue arrowhead), and pudendal nerve (yellow arrowhead). E. The rectum is separated with a mesentery at the lower part (white dashed line). F. The harvested anorectal graft with preservation of the IMA, IMV, and PNs.

## Materials and Methods

### Animals

Five healthy adult male beagle dogs weighing 7.5–15 kg were included in this study. Dog 1 was used for anatomical study and dog 2 was used as an anorectal transplantation model. Dogs 3, 4, and 5 were used for anorectal blood supply evaluation using indocyanine green (ICG) fluorescence angiography. All surgical and euthanasia procedures were performed in accordance with the “Guide for the Care and Use of Laboratory Animals” published by the National Institutes of Health (NIH Publication No. 85–23, revised 1985). The experimental protocol was approved by the Animal Experimental Committee of Kyoto University, and all efforts were made to minimize animal suffering.

### Preparation for Surgery

All surgical procedures and physiological measurements were performed under general anesthesia. The animals were premedicated by intramuscular administration of 0.05 mg/kg atropine sulfate. They were then anesthetized with 15 mg/kg ketamine hydrochloride and 3 mg/kg xylazine hydrochloride and intubated endotracheally. Continuous monitoring was performed by electrocardiography and oxygen saturation by reflectance oximetry using a sensor clipped to the ear. The abdominal and perianal regions were shaved and the animals were positioned in the supine position. The abdominal region was disinfected with 70% ethanol and iodine tincture and covered with sterilized drapes. Sevoflurane (0.5%–1.0%) and nitrous oxide gas were used for maintenance of anesthesia during the procedure, under mechanical ventilation.

### Abdominal Manipulation

Laparotomy was performed via a median incision (Fig. 1–A). The intestines were packed into the upper abdomen. The canine colorectal region resembled that of humans. The rectum was approximately 5 cm in length and 3 cm in width, and it was bilaterally supported by the levator ani muscle. The inferior mesenteric artery (IMA), anatomically known as the caudal mesenteric artery in dogs, diverged from the anterior surface of the abdominal aorta. It ramified into the anterior rectal artery (corresponding to the superior rectal artery in humans) and nourished the rectum. The inferior mesenteric vein (IMV), anatomically known as the caudal mesenteric vein in dogs, ran along the rectum. These vessels were carefully dissected using a surgical loupe (Fig. 1–B). The rectum was circumferentially dissected to the anal canal.

**Figure 2:**
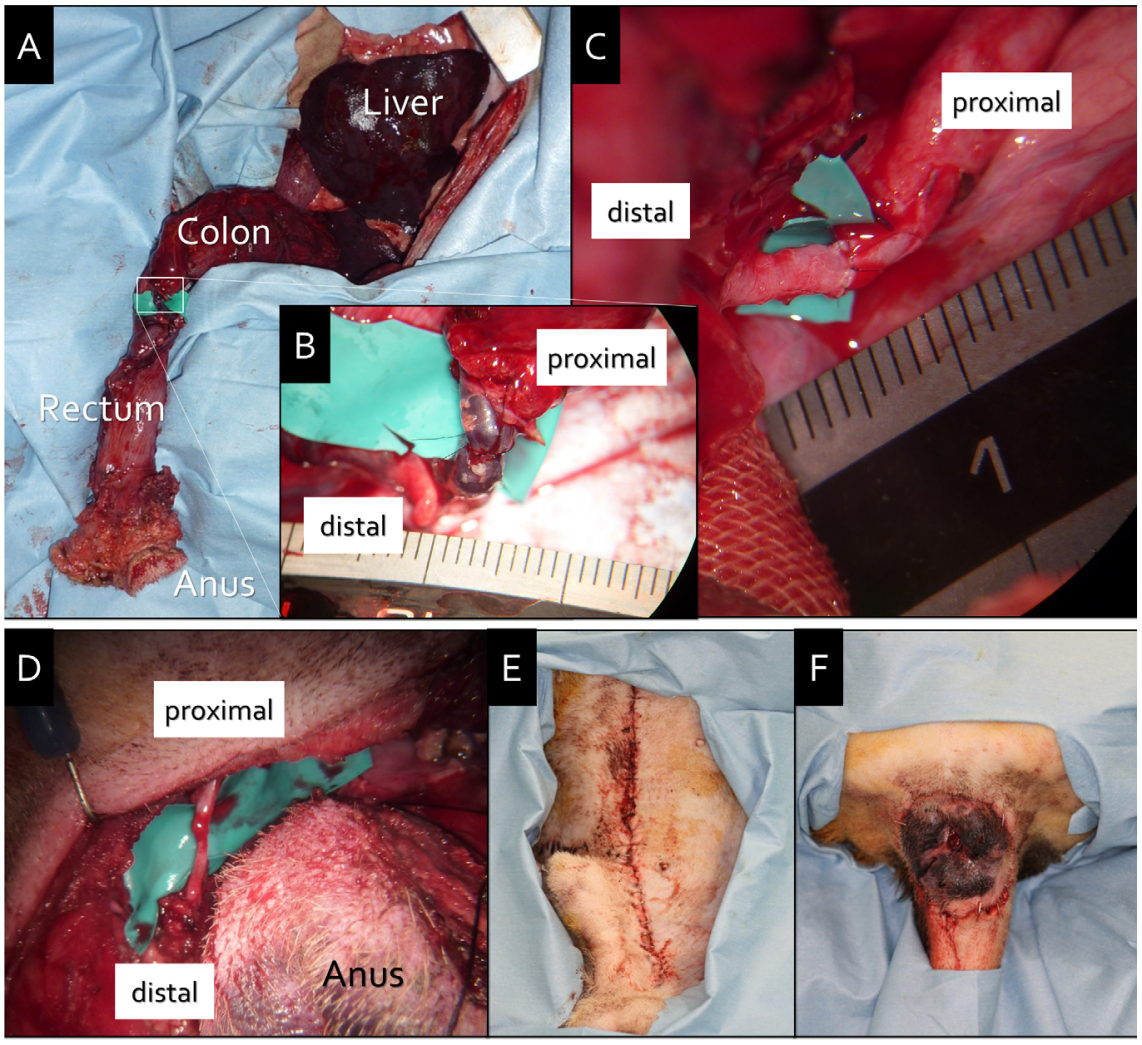
Anorectal autotransplantation. A. Extraperitoneal view after venous anastomosis. B. Magnified view of the venous anastomosis (IMV: 3.5 mm in diameter). C. Magnified view of the arterial anastomosis (IMA: 3.0 mm in diameter). D. Perineal view of the nerve anastomosis (right PN: 2.0 mm in diameter, left PN: 1.5 mm in diameter). E. Abdominal closure. F. Perineal closure; the distal end of the graft is congested.

**Table 1:** Case summary, purpose, surgical data, and used figures in all animals.

	BodyWeight	Sex	Purpose	IMAdiameter	IMVdiameter	PV diameter(right/left)	Figure
Dog1	15 kg	Male	anatomical study	3.2 mm	4.0 mm	2.0 mm/2.0mm	-
Dog2	12.5 kg	Male	autotransplantation	3.0 mm	3.5 mm	1.5 mm/2.0mm	1,2,3,4
Dog3	11 kg	Male	ICG control	–	–	–	5-A,B,C,D
Dog4	8.5 kg	Male	blood supply from the IMA	–	–	–	5-E,F,G,H
Dog5	7.5 kg	Male	blood supply from the pudendal arteries	–	–	–	5-I,J,K,L

### Perineal Resection of the Rectum

Following the circumanal incision, the anal canal was circumferentially dissected outside of the external anal sphincter muscle (Fig. 1–C). Pudendal nerves (PNs), pudendal arteries (PAs), and pudendal veins (PVs) ran bilaterally along the inside of the ischial tuberosity and reached the external anal sphincter muscle at the 2 o’clock and 10 o’clock positions, respectively (Fig. 1–D). The posterior wall of the anal segment was separated from the anterior surface of the coccygeal muscle. The levator ani muscle was transected at the lateral and posterior wall, and the anterior wall was detached behind the prostate. The rectum was separated with a mesentery at the lower part (Fig. 1–E). The IMA, IMV, PAs, PVs, and PNs were clipped and cut to avoid any damage. Finally, an anorectal graft was harvested (Fig. 1–F).

**Figure 3:**
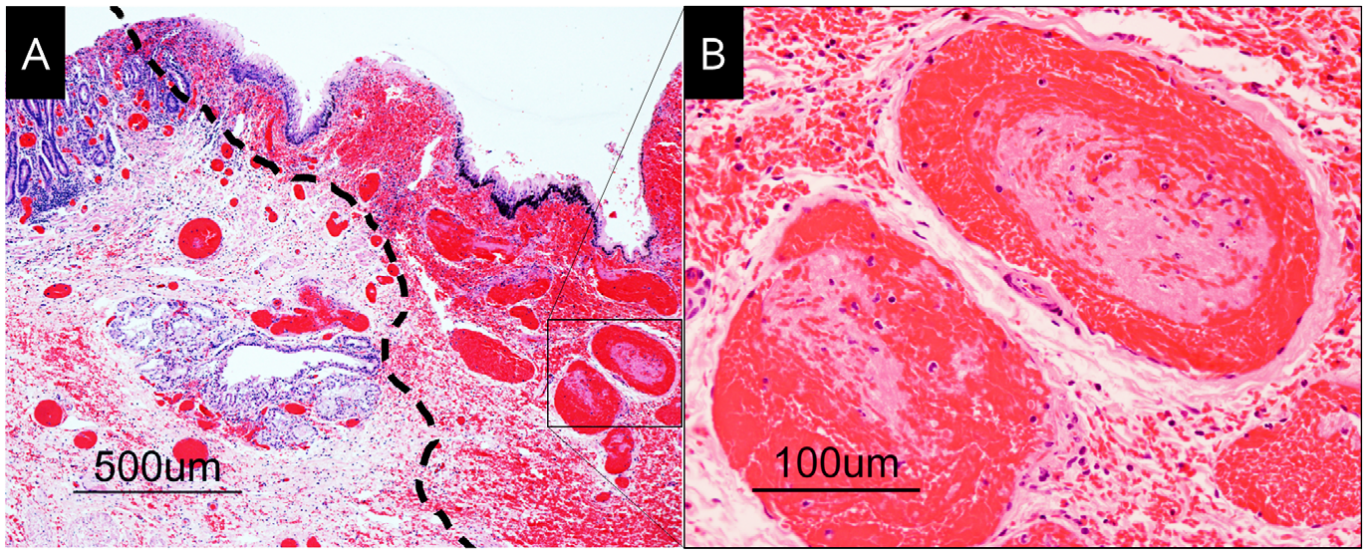
Histological findings of the graft in dog 2. A. One boundary line (black dashed line) between the congested area (right, anal side) and the normal area (left, rectum side). B. Intravascular thrombus and erosion in a magnified image of the congested area.

### Anorectal Autotransplantation

The anorectal graft was transferred to the surgical field from the back table. The rectal stump was anastomosed with a 4-0 vicryl suture (Johnson & Johnson K.K., Tokyo, Japan) using the Albert–Lambert method. All neurovascular anastomoses were performed under a surgical microscope (OME-9000, Olympus Medical Systems Corporation, Japan). First, the IMV was end-to-end anastomosed using interrupted sutures (8-0 nylon, Johnson & Johnson) outside of the abdominal cavity (Fig. 2–A, B). Then the anorectal graft was orthotopically repositioned. Second, the IMA was end-to-end anastomosed using interrupted sutures (9-0 nylon, Johnson & Johnson) within the abdominal cavity (Fig. 2–C). One artery and one vein were used for perfusion of the anorectal graft in this model. Third, the bilateral PNs were end-to-end anastomosed using epineural sutures (10-0 nylon, Johnson & Johnson) from the perineal side (Fig. 2–D). After ensuring that there were no problems with the microanastomoses or in the abdominal cavity, ileostomy was performed and the abdomen was closed (Fig. 2–E). At the perineum site, pelvic floor muscles, including the levator ani, were reconstructed using 4-0 vicryl sutures and closed (Fig. 2–F). The dogs were given liquid food after surgery, and intramuscular administration of an antibiotic (cefazolin sodium, 50 mg/kg/day) was initiated.

**Figure 4:**
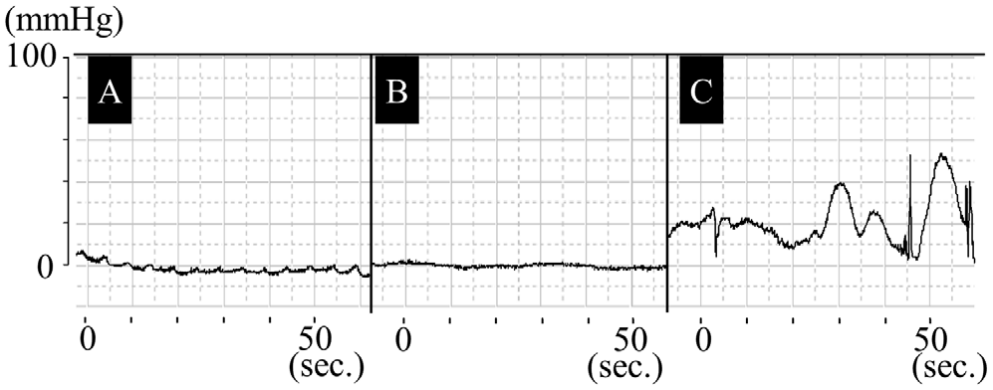
Fluctuations in anal canal continuous pressure in dog 2. A. Preoperative fluctuation in anal canal pressure. A regular rhythmic wave is observed. B. Intraoperative fluctuation in anal canal pressure. Before vascular anastomoses, the wave form is flat. C. Postoperative fluctuation in anal canal pressure. An irregular anal canal pressure wave appears after surgery.

### Functional Examination

Maximum resting pressure (highest pressure along the functional anal canal) and fluctuations in anal canal continuous pressure were examined in the lateral decubitus position using a one-channel catheter (P31-10DP, Konigsberg Instruments Inc., CA) and a measurement and analyzing system (Anorect.dll, STAR MEDICAL. INC, Japan). Examinations were performed with the rapid pull-through technique as described previously [20]. Briefly, the catheter was pulled with a speed of 10 mm/sec under anesthesia and resting conditions. Maximum resting pressure was recorded 5 times, and the average was calculated. Fluctuations in anal canal continuous pressure were measured for 5 minutes by positioning the catheter in the anal canal.

### Histological Examination

Hematoxylin and eosin-stained sections of transplanted graft tissues were pathologically evaluated. Sections were cut at several levels and meticulously examined to identify any pathological findings. The graft tissue was harvested three days after surgery, and the rectum and anal canal areas (including mucous membranes and sphincter muscles) were examined.

**Figure 5:**
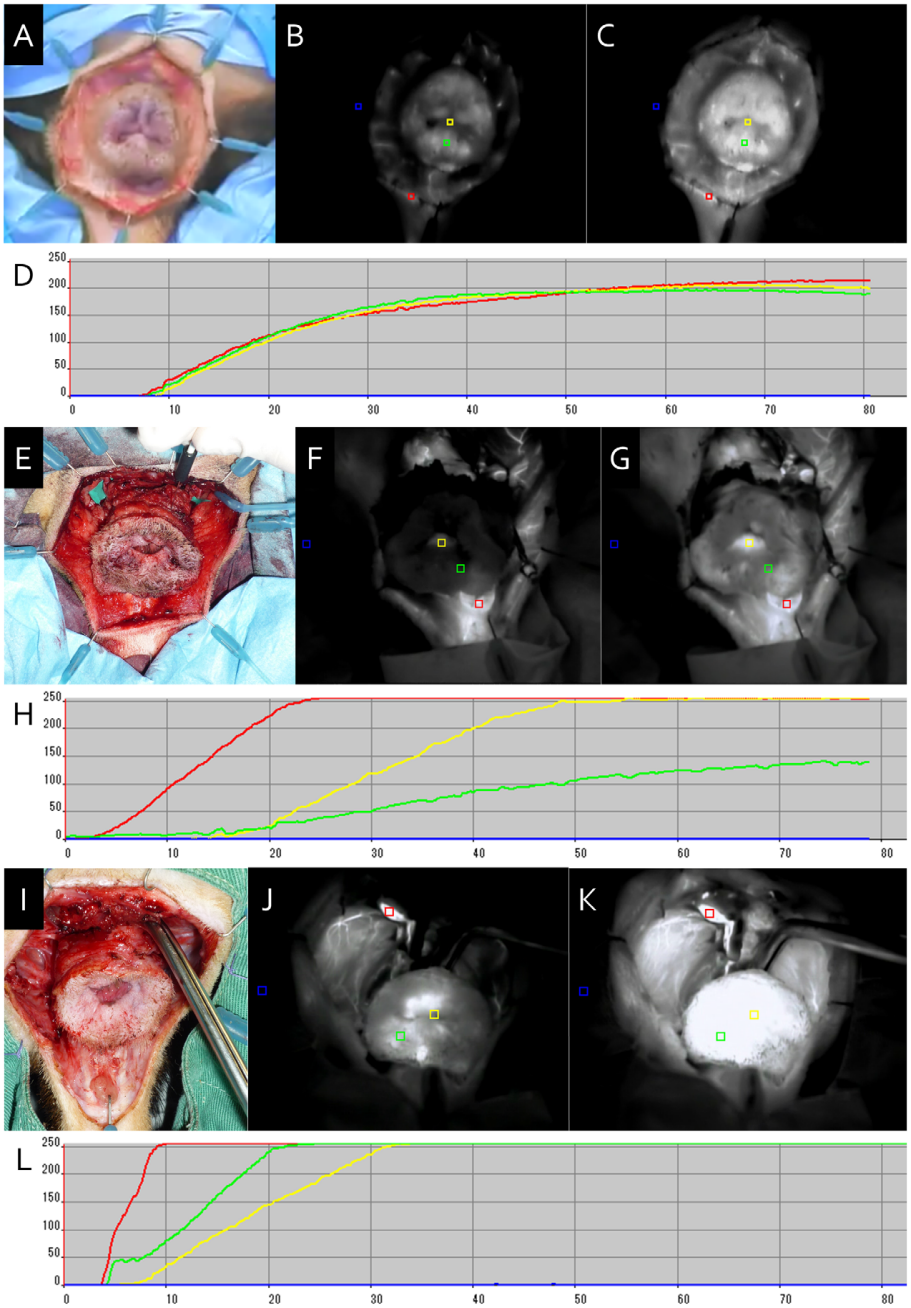
Indocyanine green fluorescence angiography of the anal blood supply A–D. Control study using dog 3. E–H. Evaluation of blood supply from the IMA in dog 4. I–L. Evaluation of blood supply from the PAs in dog 5. A, E, I. Perineal view of the anal segment immediately before angiography. B, F, J. Beginning of enhancement of the anal segment. C, G, K. Plateau of enhancement. D, H, L. Time course of brightness determined using region of interest software (red line: positive control, blue line: negative control, yellow line: anal canal, and green line: perianal skin).

### ICG Fluorescence Angiography

ICG fluorescence angiography was performed to evaluate blood supply as described previously [21], [22]. A volume of 1.0 mL ICG (0.5% diagnogreen; Daiichi Pharmaceutical, Tokyo, Japan) was intravenously injected via a peripheral venous line. Intravenously injected ICG binds to circulating globulins and remains in the intravascular region. After binding to globulins, ICG absorbs light in the near-infrared range with a maximum of 805 nm and fluoresces with a maximum of 840 nm in plasma. We utilized a newly developed near-infrared camera system (PDE-neo™; Hamamatsu Photonics KK, Shizuoka, Japan) that activates ICG with emitted light (wavelength: 760 nm). A surgeon directly handled the camera unit of the device and observed real-time images on the monitor. These videos were analyzed using region of interest software (Hamamatsu Photonics K.K., Shizuoka, Japan). Brightness was divided into a 0–250-range using the software, and the mean brightness in red (positive control), blue (negative control), yellow (anal canal), and green (perianal skin) squares was measured over time.

ICG fluorescence angiography was performed to evaluate blood supply after PA, PV, and PN dissection. Dog 3 was tested without a vessel clamp as a control. Dog 4 was tested after clamping the PAs and PVs to evaluate anal blood supply from the IMA. Dog 5 was tested after clamping the rectum to evaluate anal blood supply from the PAs.

## Results

### Surgical Procedure

Surgery was performed by a colorectal surgeon and a plastic surgeon. Dog 1 was used for a dissection study and dog 2 was used as the autotransplantation model. The duration of the autotransplantation procedure was approximately 8 hours: 3 hours for graft harvest, 2 hours for neurovascular anastomosis under a surgical microscope, and 3 hours for replantation. The vessel diameters are summarized in Table 1. The PVs formed a reticular nerve plexus near the external anal sphincter muscle and some funiculi at the exit of the pudendal canal. After vascular anastomoses, the color of the entire graft was normal. However, on procedure completion, the perianal skin color turned dark and venous congestion developed. Dog 2 recovered sufficiently to be able to walk around after the procedure; however, his appetite gradually decreased and he eventually died within three days after surgery. Just after death, samples of the anorectal graft were harvested for histological examination.

### Histological Examination

Signs of recent hemorrhage were observed around the squamocolumnar junction, and this change was observed throughout the layer of the anal canal. One boundary line (black dashed line) clearly distinguished the congested area (right, anal side) from the normal area (left, rectum side; Fig. 3–A). The congestion was much more severe in the area with squamous epithelium than in the area with columnar epithelium. Erosion was also observed; it was more severe on the anal side (right side in Fig. 3–A) than on the rectal side. Dilated and congested venous vessels were observed in the deeper area of the anal canal. Organizing thrombi were observed in the venous vessels (Fig. 3–B).

### Functional Examination

In this two-case study, the preoperative resting pressure was 30 mmHg in dog 1 and 28 mmHg in dog 2, whereas the postoperative pressure (on the day of surgery) was 22 mmHg in dog 1 and 18 mmHg in dog 2. Fluctuations in anal canal continuous pressure are presented in Figure 4. In both cases, a regular rhythm wave was recognized before surgery (Fig. 4–A). The wave during reconstructive surgery was flat before vascular anastomoses was achieved (Fig. 4–B), whereas the wave after surgery was irregular (Fig. 4–C).

### ICG Fluorescence Angiography

The IMA and PAs shared a blood supply to the anal segment. These blood flows were examined. In dog 3, anal canal and perianal skin brightness rose in a similar pattern as that in the positive control (Fig. 5–A–D; also see Video S1). In dog 4, anal canal brightness rose to >245 30 seconds after the positive control exhibited the same anal canal brightness (Fig. 5–E–H; also see Video S2) Perianal skin brightness reached a plateau at around 150. In dog 5, perianal skin brightness rose to >245 5 seconds after the positive control exhibited the same brightness (Fig. 5–I–L; also see Video S3). Ten seconds later, anal canal brightness rose to >245 in dog 5.

## Discussion

Anorectal allotransplantation was first attempted at St. Mark’s Hospital, UK in 2000 [19]. They transplanted the anorectum from female pigs to male pigs without immunosuppression and achieved successful IMA, IMV, and PN anastomosis. Histological examination revealed satisfactory inferior mesenteric flow at 24 hours after surgery; however, more detailed information regarding this is unavailable. Recently, Galvão et al. reported the development of a rat intestinal transplantation model (including the small and large bowel, rectum, and anus) by anastomoses of the recipient’s aorta to the donor’s aorta and the donor’s portal vein to the recipient’s superior mesenteric vein [17]. We have also established a rat anal autotransplantation model by anastomosing bilateral PAs and PVs [18]. This is the first report, as per our knowledge, on anorectal autotransplantation in dogs.

In this study, we confirmed the status of blood reperfusion of the graft (particularly the rectum, anal epithelium, and sphincter) after IMA and IMV anastomoses (see Video S4) with reference to a previous swine experimental model [19]. The distal side of the anorectal graft represented macroscopic venous stasis (Fig 2–F). Furthermore, histopathological examination revealed that venous congestion, rather than ischemia, was present in the area of the anal canal (Fig. 3). The area drained by the IMV was intact, whereas that drained by the PVs was congested and necrotized. We performed ICG fluorescence angiography to investigate the anal blood supply in dogs 3, 4, and 5. The IMA supplied sufficient blood to the anal canal; however, its supply to the perianal skin was inadequate. Meanwhile, the PAs supplied enough blood to both the anal canal and the perianal skin. Although this result was limited to arterial blood flow, the PVs were also expected to have better venous flow than the IMV. From the results of histological examination and ICG angiography, we conclude that the bilateral PAs and PVs are ideal vascular pedicles in an anorectal transplantation model in beagle dogs.

In the functional examination, we identified decreased resting pressure and a postoperative irregular anal canal pressure wave, which was not evident before or during surgery (Fig. 4). This wave pattern refers to the rectal automatic movement caused by graft revascularization and lack of sphincter control. Nevertheless, issues concerning sphincter control as well as immune rejection still remain. A transplant model that can be evaluated over a longer term is thus required.

Dogs are as suitable as humans for the observation of defecation control. They can endure defecation desire and defecate at appropriate places as a result of training. In addition, they have vessel sizes and hemodynamic parameters that approximate those of human vessels [23]. Therefore, performing such experiments in a canine model is both necessary and sufficient for the study of anal reconstruction.

In this study, blood supply to the anal segment, which was essential to anorectal transplantation, was shown. However, some issues remained unresolved. First, realistic clinical applications for anorectal transplantation remain controversial. According to O’Bichere et al., the prospect of anorectal amputation for cancer and a permanent colostomy is so disturbing for patients that they would prefer death to treatment and cure. For such patients, the prospect of clinical anorectal transplantation may be considered lifesaving [19]. Moreover, the common diseases are congenital anal dysfunction caused by anal atresia or Hirschsprung’s disease and intractable anal fistula due to inflammatory bowel diseases, trauma, and rectal or perianal cancer. These are not considered as indications for regenerative medicine. Second, functional recovery after autotransplantation remains controversial. Anal function is very complicated, and vascularized composite tissue allotransplantation (VCA) is considered the only method for reconstructing organs with complicated functions, such as the face [14] and hands [15]. Long-term follow-up studies are required to confirm this. Third, monitoring for infection and graft rejection remains a questionable issue. Transplantation of the colon along with the intestinal allograft was previously avoided because of the risk of infection [24], but it is sometimes performed in the present date [25], [26]. Approximately 80% immune cells normally reside in the gut, and they are repopulated after transplantation with recipient cells [27]. Further allotransplantation studies involving immunosuppressants and antibiotics are required to reveal new information concerning immunogenicity of the transplanted anus.

There has been significant progress in the field of transplantation medicine in recent years. However, research into anal transplantation has been delayed because the field encompasses various areas of study, such as colorectal surgery, pediatric surgery, plastic surgery, and transplantation medicine. Our findings may help in further advancement of research on anorectal transplantation.

## Supporting Information

Video S1 **ICG fluorescence angiography to evaluate normal anal blood supply in dog 3.**
(WMV)

Video S2 **ICG fluorescence angiography to evaluate anal blood supply from the IMA in dog 4.**
(WMV)

Video S3 **ICG fluorescence angiography to evaluate anal blood supply from the PAs in dog 5.**
(WMV)

Video S4 **Venous reperfusion of the graft after replantation in dog 2.**
(WMV)
